# From aging to Alzheimer's disease: concordant brain DNA methylation changes in late life

**DOI:** 10.1002/alz70856_107369

**Published:** 2026-01-09

**Authors:** Lily Wang, David Lukacsovich, Juan I Young, Lissette Gomez, Michael A. Schmidt, Wei Zhang, Brian W Kunkle, X. Steven Chen, Eden R. Martin

**Affiliations:** ^1^ University of Miami Miller School of Medicine, Miami, FL, USA; ^2^ Department of Public Health Sciences, University of Miami Miller School of Medicine, Miami, FL, USA; ^3^ John P. Hussman Institute for Human Genomics, University of Miami Miller School of Medicine, Miami, FL, USA; ^4^ Dr. John T. Macdonald Foundation Department of Human Genetics, University of Miami Miller School of Medicine, Miami, FL, USA

## Abstract

**Background:**

Aging is a major risk factor for Alzheimer's disease (AD), but the molecular processes linking aging to AD remain unclear. Epigenetic modifications, particularly DNA methylation (DNAm), play a crucial role in understanding aging and AD.

**Method:**

We studied brain DNA methylation (DNAm) changes in normal aging versus AD in late life. We performed a comprehensive meta‐analysis of two large cohorts of postmortem prefrontal cortex samples from subjects over 65 years old. Our analysis adjusted estimated cell‐type proportions (i.e., the proportion of neurons), sex, and batch effects, and corrected for inflation and multiple testing.

**Result:**

We identified numerous DNAm differences consistently associated with aging in both cohorts, highlighting key genes such as *ELOVL2*, *ISM1*, and *KLF14*, which are implicated in various aging processes. These DNAm differences are predominantly hypermethylated, enriched in promoter regions, and associated with genes involved in immune processes and metabolic functions. Our results also revealed significant overlaps between aging‐associated DNAm differences and those involved in AD, supporting the hypothesis that aging and AD are interconnected at the molecular level. Intriguingly, nearly all DNAm differences significantly associated with both age (at death) and AD Braak stage showed concordant effect sizes in the same direction.

**Conclusion:**

Our study provides valuable insights into the aging‐associated epigenetic landscape and its potential implications for AD. As aging and AD are intertwined, targeting age‐related epigenetic modifications may offer new therapeutic strategies for AD.

